# Evaluating Seismocardiography as a Non-Exercise Method for Estimating Maximal Oxygen Uptake

**DOI:** 10.3390/healthcare12212162

**Published:** 2024-10-30

**Authors:** Robert Schulenburg, Samuel Emil Schmidt, Jan Schröder, Volker Harth, Rüdiger Reer

**Affiliations:** 1 Department of Sports and Exercise Medicine, Institute of Human Movement Science, University of Hamburg, 20148 Hamburg, Germany; jan.schroeder@uni-hamburg.de (J.S.); ruediger.reer@uni-hamburg.de (R.R.); 2 Institute for Occupational and Maritime Medicine Hamburg (ZfAM), University Medical Center Hamburg-Eppendorf (UKE), 20251 Hamburg, Germany; harth@uke.de; 3 Department of Health Science and Technology, Aalborg University, 9220 Aalborg Øst, Denmark; sschmidt@hst.aau.dk

**Keywords:** cardiorespiratory fitness, maximal oxygen uptake, VO_2MAX_, seismocardiography, non-exercise estimation of VO_2MAX_, machine learning

## Abstract

Background: The value of maximal oxygen uptake (VO_2MAX_) is a key health indicator. Usually, VO_2MAX_ is determined with cardiopulmonary exercise testing (CPET), which is cumbersome and time-consuming, making it impractical in many testing scenarios. The aim of this study is to validate a novel seismocardiography sensor (Seismofit^®^, VentriJect DK, Hellerup, Denmark) for non-exercise estimation of VO_2MAX_. Methods: A cohort of 94 healthy subjects (52% females, 48.2 (8.7) years old) were included in this study. All subjects performed an ergometer CPET. Seismofit^®^ measurements were obtained 10 and 5 min before CPET in resting condition and 5 min after exhaustion. Results: The CPET VO_2MAX_ was 37.2 (8.6) mL/min/kg, which was not different from the two first Seismofit^®^ estimates at 37.5 (8.1) mL/min/kg (*p* = 0.28) and 37.3 (7.8) mL/min/kg (*p* = 0.66). Post-exercise Seismofit^®^ was 33.8 (7.1) mL/min/kg (*p* < 0.001). The correlation between the CPET and the Seismofit^®^ was r = 0.834 and r = 0.832 for the two first estimates, and the mean average percentage error was 11.4% and 11.2%. Intraclass correlation coefficients between the first and second Seismofit^®^ measurement was 0.993, indicating excellent test-retest reliability. Conclusion: The novel Seismofit^®^ VO_2MAX_ estimate correlates well with CPET VO_2MAX_, and the accuracy is acceptable for general health assessment. The repeatability of Seismofit^®^ estimates obtained at rest was very high.

## 1. Introduction

The value of maximal oxygen uptake (VO_2MAX_) in the fields of cardiorespiratory fitness (CRF), health, disease, and exercise science has been established through numerous investigations and is a very important term in sports medicine. In exercise physiology, peak oxygen consumption (VO_2PEAK_) is an important term, which connects the interplay between the pulmonary, cardiovascular, and muscular systems in transporting oxygen from the atmosphere to the working muscles [1]. Multiple studies over the past 3 decades have shown increasing evidence that low levels of CRF are associated with a high risk of cardiovascular disease (CVD) and higher mortality. Recent studies have demonstrated that a low fitness level carries a higher risk than any other cardiac risk factors such as smoking, diabetes, hypertension, known cardiac disease, atrial fibrillation, and chronic kidney disease [2]. Furthermore, lower CRF levels are related to various cancers, especially of the breast and the digestive tract [3,4,5,6]. The encouraging results are that even moderate improvement in fitness leads to significant mortality reduction [7]. Echoing these statements, in 2016 the American Heart Association (AHA) declared that CRF is important for CVD risk prediction [8]. It can be concluded that increased CRF by itself leads to both reduced mortality and reduced morbidity.

This underscores the crucial role fitness plays in contemporary health and medical practices, which was underlined in the editorial by Kaminsky et al. in the *Journal of the American College of Cardiology*, which encouraged both clinicians and public health professionals to adopt CRF as a key health indicator [9]. Traditionally, VO_2MAX_ is ascertained using ‘Cardiopulmonary Exercise Testing’ (CPET) combined with spiroergometry, carried out under maximum exhaustion in diagnostic laboratories. However, this method is laborious and time-intensive, rendering it less feasible for numerous testing situations.

Seismocardiography (SCG) is an emerging non-invasive technology that measures cardiac function from cardiac-induced vibrations at the chest wall, using an accelerometer [10,11]. Multiple studies have linked the SCG signal to cardiac function [12,13]. It is well-established that the heart’s maximal cardiac output is the dominating component of VO_2MAX_ [14]. Sørensen et al. described a correlation between resting SCG measures and VO_2MAX_ [15], and Hansen et al. validated early-stage prototypes for a non-exercise SCG-based VO_2MAX_ estimation [16].

A non-exercise method for estimating CRF could lower the barriers related to CRF estimation in clinical settings and thus utilize CRF as a common clinical biomarker. Therefore, this study aims to evaluate the Seismofit^®^ (VentriJect DK), a novel medical device that employs SCG to estimate VO_2MAX_, by comparing its results to those derived from a cycle ergometer CPET. In addition, we test the repeatability and robustness of the device and compare it to other CRF estimation methods.

## 2. Methods

A heterogeneous group of 107 healthy subjects was recruited for this study. To be able to conduct sub-group analyses, we aimed to recruit at least 90 subjects. Exclusion criteria were the following: Chronic diseases in general, high blood pressure (≥130/90), orthopaedic pre-existing conditions, acute illness, and pre-exhaustion failure of the spiroergometry as defined below. Beforehand, subjects were asked to do no more than light physical exertion in the 2 days before the CPET. On the day of the test itself, any physical activity should have been avoided. Furthermore, all participants underwent a medical examination prior to the CPET, a medical history was taken of any pre-existing conditions and medication taken, it was determined how much regular endurance sport is practised, their daily weight and height were measured, and a resting ECG was written. To minimize anxiety, the test procedures were explained adequately, and the test environment was calm and private. The CPET took place in an exercise physiology laboratory at the Faculty of Human Movement Science, University of Hamburg.

The study protocol was approved by the local scientific ethical committee. Subjects signed a written informed consent before participating in the study, and the study complies with the Declaration of Helsinki.

### 2.1. Cardiopulmonary Exercise Testing

Participants performed an incremental ramp protocol (RAMP Test) until voluntary exhaustion on a mechanically braked cycle (Ergoselect 4 SN, Ergoline GmbH, Bitz, Germany). After initial 3 min at 50 Watts (W), the cycling power consistently increased by 1 W every 3.6 s (equivalent to 50 W/3 min). After termination of the CPET, the protocol provided a 3-min cool-down phase on the cycle ergometer at a load of 50 watts. Michalik et al. described the RAMP Test (~0.277 W/s) compared to the STEP Test (50 W/min) to determine VO_2MAX_, peak power output, and ventilatory thresholds more precisely [17].

ECG was recorded continuously (12-channel ECG CardioPart 12 Blue) with a sampling rate of 500 Hz (AMEDTEC Medizintechnik Aue GmbH, Aue, Germany) using a desktop software (AMEDTEC ECGpro version 5.10.002). Gas exchange kinetics were recorded with a metabolic analyser in breath-by-breath mode (Quark CPET, module A-67-100-02, COSMED Germany GmbH, Fridolfing, Germany; desktop software: Omnia version 1.6.5). The breath-by-breath method measures the flow, the oxygen concentration, and carbon dioxide concentration time dependently with gas analyzers with a typical frequency (100 Hz) much higher than the maximum breathing frequency (~1 Hz).

Gas calibration of the Quark CPET was carried out before each test according to the manufacturer’s instructions for use. Spiroergometric tests of the individual subjects were conducted at different times and at different room temperatures due to the laboratory capacity and availability of the subjects. We ensured to follow the guidelines recommended by the manufacturer. All measurements have been carried out in accordance with the manufacturer’s instructions at a temperature range of 10 °C to 40 °C. Additionally, sufficient room ventilation was ensured at all times to avoid any measurement errors regarding gas analyses.

Regarding the ACSM (American College of Sports Medicine) guidelines [18], the protocol was terminated when: (A) the subject could not hold the predetermined cadence (60 rpm), (B) the subject reached the Top level of the Borg Rating of Perceived Exertion Scale [19], or (C) due to self-determination. Exhaustion was assumed when the following criteria were fulfilled: (A) heart rate > 90% of the maximum predicted heart rate (prediction model according to Tanaka et al. [20]: 208 − (0.7 × age), and (B) respiratory quotient > 1.05. Maximum oxygen uptake (VO_2MAX_) and maximum HR (HR_MAX_) were defined as the average VO_2_ and HR over the last 30 s of the test.

### 2.2. Seismocardiography Measurements

**Figure 1:**
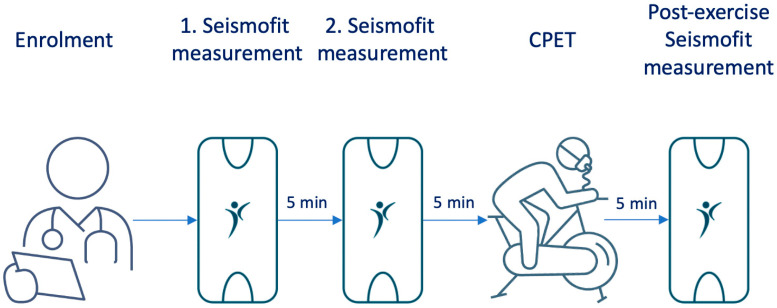
The study protocol. After enrolment two Seismofit^®^ recordings were obtained with 5-min intervals before the CPEP test. A third Seismofit^®^ recording was obtained 5 min after the CPET test.

**Figure 2:**
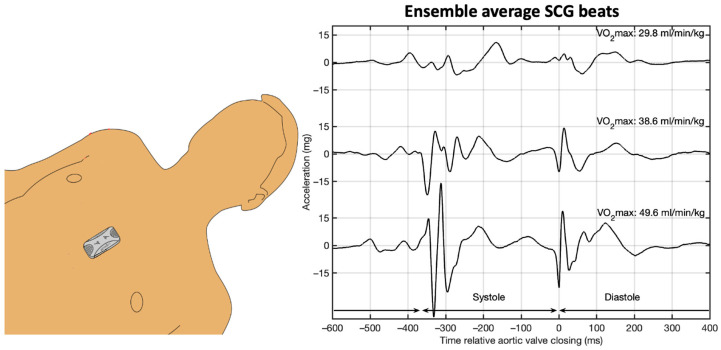
**Left**: The Seismofit^®^ mounted at the lower sternum in supine position. **Right**: Ensemble average SCG’s from three males with different spirometry VO_2MAX_ levels.

The Seismofit^®^ device is a small accelerometer sensor (50 mm × 30 mm × 15 mm) that is controlled by a smartphone app. We conducted measurements using the Seismofit^®^ device at three time points: 10 min and 5 min prior to spiroergometry under resting conditions, and to quantify the influence of non-resting conditions 5 min after exhaustion (Figure 1). The Seismofit^®^ sensor was positioned at the lower sternum using a double adhesive patch (Figure 2) and the recording time was 42 s, while the subject was in a supine position. A smartphone app was utilized to control the Seismofit^®^ sensor, and the recorded seismocardiography (SCG) data were automatically transmitted to a cloud server for processing.

The processing of SCG recordings was performed by VentriJect DK using algorithm version 4.7 [21]. VentriJect was blinded to the spiroergometric results of the current study. The algorithm employed the ensemble average SCG heartbeat (see Figure 2) and employed machine learning techniques to estimate VO_2MAX_ from the SCG beat, in combination with anthropometric data, gender, age, height, and weight. The regression Equation (1) includes four SCG-derived measures: frequency of the systolic SCG complex quantified using principal component analysis (Sys_Spectrum_), the morphology of the diastolic SCG complex quantified using principal component analysis (Dia_Morphology_), peak to peak amplitude of the average SCG diastolic complex (Dia_Peak to Peak_), and the heartbeat duration (RR):

(1) $${VO}_{2max}\sim \omega_{0}+\omega_{1}\;Sex+\omega_{2\;}Age+\omega_{3}Weight+\omega_{4}Height+\omega_{5}RR+{\omega_{6}Dia}_{Peak\;to\;Peak}+\omega_{7}{\;Dia}_{Morphology}+\omega_{8}{\;Sys}_{Spectrum}$$

Dia_Morphology_ and Dia_Peak to Peak_ quantify the heart’s diastolic function. Agam et al. demonstrated that Dia_Peak to Peak_ correlated to the echocardiographic e’ parameter, which is related to diastolic filling [22]. Sys_Spectrum_ aims at quantifying the heart’s contractability. Sørensen et al. demonstrated the systolic SCG complexes is altered by bi-ventricular pacing. The correlation between the individual equation compotes and VO_2MAX_ is found in Table A1 in Appendix A.

### 2.3. Benchmarking Against Other Methods

We compared the performance of the Seismofit^®^ to the non-exercise algorithm from the FRIENDS study [23]:

*VO*_2_*max* ~ 79.9 − (0.39 × *age*) − (13.7 × *sex* [0 = *men*; 1 = *women*]) − (0.127 × *weight* [*lbs*]) (2)

In addition, we compared to the ACSM metabolic equation where VO_2MAX_ is estimated from the peak power output [18]:

VO_2_max ~ 10.8 × Peak Power Output/Weight + 7 (3)

### 2.4. Statistical Analysis

Variables were expressed as mean (standard deviation), while categorical variables were reported as frequencies (percentages). Paired Student’s *t*-tests were used for comparisons between VO_2MAX_ estimates, and Pearson’s tests were used to analyze correlations between variables. All continuous variables were validated as approximately normally distributed via visual analysis. The interpretation of Pearson correlation coefficients followed the classification: very high (>0.90), high (0.70–0.90), moderate (0.50–0.70), low (0.30–0.50), and irrelevant (0.00–0.30) [24].

Estimation error was assessed using the mean average percentage error (MAPE) and standard error of estimate (SEE). Bias, SEE, and MAPE were expressed with their respective [95% confidence intervals]. Repeatability was measured using intraclass correlation coefficients (ICC) with absolute agreement, MAPE, and root mean square (RMS) of the difference between the scores. The Steiger method was employed to test for differences between two correlation coefficients [25]. Additionally, a paired *t*-test of average percentage errors was conducted to assess statistical significance between MAPE values of different scores.

For detailed analyses of specific subgroups, separate analyses were performed for males and females, adults younger or older than 50 years, recreational physically active (exercising for more than 5 h per week), or sedentary subjects, and subgroups classified by BMI (BMI < 25 kg/m^2^: normal weight, BMI 25 to 29.9 kg/m^2^: overweight, BMI ≥ 30 kg/m^2^: obese). Additionally, subgroups were classified based on VO_2MAX_ levels as follows: low fitness level (VO_2MAX_ < 30 mL/kg/min), moderate fitness level (VO_2MAX_ 30 to 44.9 mL/kg/min), and high fitness level (VO_2MAX_ ≥ 45 mL/kg/min).

## 3. Results

A heterogeneous group of 107 healthy subjects was recruited for this study. Afterwards, 11 subjects of this study population were excluded due to the premature termination of spiroergometry, and two subjects were excluded since the battery level at the device was too low to obtain either of the Seismofit^®^ recordings. The remaining 94 subjects were 52.1% females, 48.2 (8.7) years old, with a BMI of 24.7(3.6) kg/m^2^ and 50% with an active lifestyle, Table 1. The CPET VO_2MAX_ was 37.2 (8.6) mL/min/kg, which was 14.5% higher than the VO_2MAX_ of 32.5 (6.8) mL/min/kg predicted by the non-exercise algorithm from the FRIENDS study, indicating that the subjects were slightly fitter than the background population. After the exclusion of the subjects with premature termination of spiroergometry, the correlation between max watt and unadjusted VO_2_max was very good (r = 0.947), validating the quality of the CPET measurement.

### 3.1. Seismocardiographic Estimation of VO_2MAX_

The first Seismofit^®^ measurements were successfully obtained in 93 out of the 94 subjects, and the second Seismofit^®^ measurements were also successfully obtained in 93 out of the 94 subjects. Post-exercise Seismofit^®^ measurements were obtained in 92 subjects. The reason for not obtaining Seismofit^®^ measurements was low device battery power.

**Figure 3:**
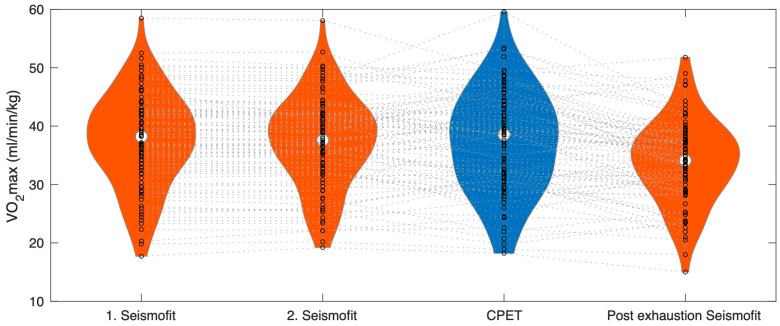
Vilon plot of the VO_2MAX_ values estimated by Seismofit^®^ and CPET. The white circle represents means. Seismofit^®^ and Scatter plots of Seismofit^®^ and CPET VO_2MAX_ from the first and second Seismofit^®^ measurements prior to exercise and the third measurement post-exercise.

The first pre-exercise Seismofit^®^ measurement yielded a value of 37.5 (8.1) mL/min/kg, and the second Seismofit^®^ measurement yielded a value of 37.3 (7.8) mL/min/kg (see Table 1 and Figure 3). However, neither the first nor the second Seismofit^®^ measurements showed a significant difference compared to the CPET VO_2MAX_ at 37.2 (8.6) mL/min/kg (*p* = 0.64 and *p* = 0.96, respectively). The average of the two first Seismofit^®^ measurements was 37.4 (8.0) mL/min/kg.

After exercise, the Seismofit^®^ score dropped to 33.8 (7.1) mL/min/kg, which was significantly different from both the pre-exhausting Seismofit^®^ estimates and the CPET estimate (*p* < 0.001).

**Figure 4:**
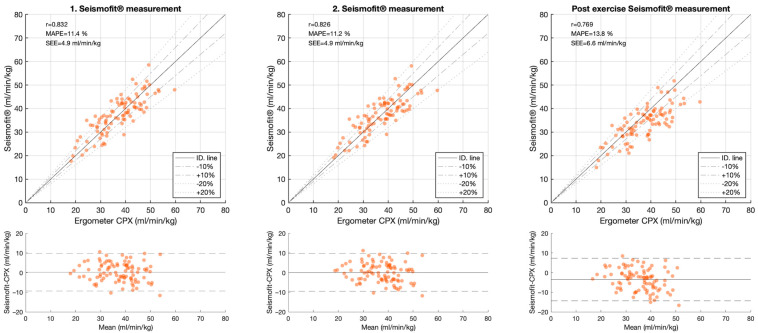
Top row: Scatter and Bland Altman plots between Seismofit^®^ and CPET VO_2MAX_ from the first and second Seismofit^®^ measurement prior to exercise and the third measurement post exercise. Bottom row: Bland Altman plots including 95% lines of agreement.

The correlation between the CPET and the Seismofit^®^ measurements was high; with r = 0.834 [0.76, 0.89] for the average estimate, r = 0.832 [0.76, 0.89] for the first measurement, r = 0.826 [0.75, 0.88] for the second measurement, and r = 0.769 [0.67, 0.84] for the post-exercise measurement, Table 2 and Figure 4. MAPE was 11.4% [9.74, 13] for the first estimate, 11.2% [9.59, 12.9] for the second estimate, and 13.8% [12, 15.6] for the post-exercise estimate. The limits of the agreement were −9.3 to 9.8 mL/kg/min in the first measurement and −9.3 to 9.8 mL/kg/min in the second measurement.

### 3.2. Repeatability

Repeatability was determined by the two subsequent pre-exercise measurements. Intraclass correlation coefficients indicated excellent test-retest reliability between the two first recordings with ICC value at 0.993. MAPE between the first and second recordings was 1.87%, and RMS was 0.93 mL/min/kg.

### 3.3. Comparison to Other Estimation Methods

The estimation error of the FRIENDS non-exercise VO_2MAX_ estimation algorithm was found to be MAPE 15.2% [13.1, 17.4], which was larger than the error of the pre-exercise Seismofit^®^ estimations (*p* < 0.001). Additionally, the correlation between the FRIENDS algorithm and CPET was 0.752 [0.65, 0.83], demonstrating inferior performance compared to the Seismofit^®^ (*p* = 0.003), see Table 2.

On the other hand, the ACSM MaxWatt method showed a higher correlation with CPET, with a correlation coefficient of 0.947 [0.92, 0.96], surpassing that of the Seismofit^®^ (*p* < 0.001). However, the ACSM MaxWatt method overestimated VO_2MAX_, with a mean overestimation of 7.1 [6.5, 7.7] ml/min/kg. Consequently, the MAPE for ACSM MaxWatt was 20.6% [18.4, 22.7], which was inferior to that of the Seismofit^®^ (*p* < 0.001).

### 3.4. Subgroups Analysis

Subjects with an active lifestyle did have a significantly higher CPET VO_2MAX_ (42.4 (7.1) mL/min/kg) than subjects with a sedentary lifestyle (32.0 (7.1) mL/min/kg) (*p* < 0.001). This was also observed by the Seismofit^®^, which estimated VO_2MAX_ significantly higher in subjects with an active lifestyle (41.2 (6.3) mL/min/kg) compared to those with a sedentary lifestyle (33.7 (7.7) mL/min/kg) (*p* < 0.001), Table 1. However, the Seismofit^®^ tended to overestimate VO_2MAX_ in subjects with a sedentary lifestyle, with a mean overestimation of 1.5 [0.25, 2.8] mL/min/kg, Table 3. Conversely, it tended to underestimate VO_2MAX_ in subjects with an active lifestyle, with a mean underestimation of −1.4 [−2.9, −0.003] ml/min/kg. Similarly, the Seismofit^®^ showed overestimation in subjects with a low fitness level (2.7 [0.78, 4.6] mL/min/kg), corresponding to 10.7%, and underestimation in subjects with a high fitness level VO_2MAX_ (−3.4 [−5.9, −0.89] mL/min/kg), corresponding to −6.7%. When examining the results based on gender, age groups, and weight groups, no significant bias was observed, Table 3.

## 4. Discussion

VO_2MAX_ is a key health indicator, with a high prognostic value. However, its accessibility remains limited in many health and clinical settings. In this study, we validated the Seismofit^®^, an innovative device designed to estimate VO_2MAX_ without the need for exercise in both sedentary and active subjects.

### 4.1. VO_2MAX_ Estimation

The correlation between the Seismofit^®^ and the CPET was high in both the first and second recordings (r = 0.832 & r = 0.826), and no estimation bias was observed. The estimation error (SEE = 4.9 mL/min/kg & MAPE 11.2–11.4%) of the device is acceptable for many applications in health assessment. However, there is no clear definition of what an acceptable estimation error is. Hansen et al. recommended a MAPE < 10% [16], which was not fully met in the current study. Meanwhile, Molina-Garcia et al. from the INETLIVE network [26] argue that clinical tests should be able to detect a change in VO_2MAX_ of 1.75–3.5 mL/min/kg, since clinical studies have demonstrated that an increase of 1.75 to 3.5 mL/min/kg has significant health benefits. It is therefore important that the repeatability of the Seismofit^®^ was very high (ICC = 0.993) with a very low variation between the first and the second measurements (RMS = 0.93 mL/min/kg). The trade-off between the more accurate CPET test and more feasible VO_2MAX_ estimation methods like the Seismofit^®^ depends on multiple factors. These include the availability of CPET equipment, whether the subject can exercise to exhaustion, safety concerns, and the time and resources available for testing.

Compared to the prior prototype algorithm version 4.3 reported by Hansen et al., the current algorithm version 4.7 had a higher correlation (r = 0.832 & r = 0.826 vs. r = 0.73) and a lower estimation error (SEE = 4.9 mL/min/kg vs. SEE = 5.9 mL/min/kg).

### 4.2. Comparison to State-of-the-Art Estimation Methods

The Seismofit^®^ was superior to the non-exercise algorithm from the FRIENDS study, in both correlation and estimation error. In comparison with other non-exercise VO_2MAX_ tests described in the scientific literature, such as wearable watches, the estimation error of the Seismofit^®^ was also substantially lower. Molina-Garcia et al. reported that the limits of agreement of non-exercise watches were −13.97 to 17.41 mL/kg/min, while the limits of agreement of the first Seismofit^®^ recording were −9.3 to 9.8 mL/kg/min, positioning the Seismofit^®^ as potentially the most accurate non-exercise estimation method.

The correlation between the ACSM MaxWatt method and the CPET was higher than the correlation between the Seismofit^®^ and the CPET, but the ACSM equation overestimated VO_2MAX_ with 7.1 mL/min/kg, leading to a high estimation error at 20.6% MAPE. However, if this systematic error is accounted for and exercise to exhaustion is feasible, the MaxWatt method can provide more accurate estimates of VO_2MAX_ than the current Seismofit^®^ method.

An alternative to MaxWatt tests is the sub-maximal test, which requires some level of exercise. Here, the heart rate at one or two load levels of exercise is used to extrapolate to a VO_2MAX_. Commonly used tests are the Aastrand and the YMCA test. Beekley et al. validated the YMCA test and found a correlation at r = 0.79 between ergometer CPET and the YMCA estimate [27]. Their measure of total error corresponds to our SEE measure. The total error of the estimate of the YMCA test was 18.5 mL/min/kg, which is considerably higher than the SEE of Seismofit^®^ at 4.9 mL/min/kg. Similar performance was identified for the Aastrand test with r = 0.71–0.78 and SSE 6.2–9.7 mL/min/kg [28].

### 4.3. Use of the Seismofit^®^

The Seismofit^®^ offers a swift, straightforward, and objective method for estimating VO_2MAX_ with notable accuracy. This could ease access to VO_2MAX_ during health evaluations, lifestyle interventions, risk assessments, and the monitoring of athletic performance.

In our study, we found no significant variance in estimation error across gender, weight categories, or age groups. This suggests that the Seismofit^®^ has a broad application scope within a generally healthy demographic. The Seismofit^®^ distinctly estimated higher VO_2MAX_ values in active subjects compared to their sedentary counterparts, illustrating its capability to discern between the effects of different lifestyles. However, the data also indicates that the Seismofit^®^ tends to underestimate VO_2MAX_ in high-fitness-level individuals and individuals with active lifestyles and overestimate it in those with a low-fitness-level VO_2MAX_ and a sedentary lifestyle. Further model developments should focus on improving estimation accuracy, especially for individuals with sedentary lifestyles who can’t perform graded exercise testing. These individuals might have the largest benefit of cardiovascular risk estimation.

Post-exercise, the Seismofit^®^ estimates were lower than the two pre-exercise estimates (33.8 mL/min/kg vs. 37.4 mL/min/kg). This suggests that the optimal time to gauge VO_2MAX_ using the Seismofit^®^ is when the individual is at rest and relaxed. Consequently, we do not recommend using the Seismofit^®^ to estimate VO_2MAX_ immediately after physical exertion, as this can skew the results.

### 4.4. Study Limitations

One significant limitation of this study is that 11 subjects did not reach exhaustion and were consequently excluded from the analysis. Reasons for not reaching the maximum load were failure of the CPET equipment, software failure, muscular cramps, anxiety, sudden pain events, not reaching a heart rate > 90% of the maximum predicted heart rate (prediction model according to Tanaka et al. [20]: 208 − (0.7 × age), or a respiratory quotient < 1.05. Furthermore, we excluded individuals based on several criteria: the presence of chronic diseases, hypertension, orthopaedic pre-existing conditions, and acute illnesses. This restricts our findings primarily to populations without these diagnoses. It is also important to note that since Seismofit^®^ estimates are derived from cardiac signal analysis, the respiratory and metabolic components of VO_2MAX_ are not considered in the Seismofit^®^ estimation. Last, the various CPET measurements were not carried out under exactly the same laboratory conditions. At least they differed in time of day, temperature, and humidity. In addition, there was no further CPET testing to validate and refer the measured VO_2MAX_ values.

### 4.5. Study Strength and Further Studies

The strength of this study lies in the validation of the Seismofit^®^ within a heterogeneous healthy population, encompassing a wide age span and individuals with varying levels of activity and fitness. Future research should focus on validating this tool in specific patient groups, such as cardiac patients and those undergoing major surgery or cancer treatment, who could benefit from VO_2MAX_ estimation for clinical treatment planning.

## 5. Conclusions

The Seismofit^®^ presents a novel and reliable approach to estimating VO_2MAX_ without exercise. This innovative method can potentially pave the way for broader utilization of VO_2MAX_ as a key health indicator. Further research should focus on model calibration and validation for subjects with very poor fitness levels where the capability to perform graded exercise testing is very limited.

## Figures and Tables

**Table 1:** Baseline characteristics of included subjects.

	All	Sedentary Lifestyle	Active Lifestyle
n	94	47	47
Female sex (*p* = 0.063)	49 (52.1%)	29 (61.7%)	20 (42.6%)
Age (years) (*p* = 0.43)	48.2 (8.7)	48.9 (9.7)	47.5 (7.6)
Height (m) (*p* = 0.081)	1.8 (0.08)	1.7 (0.09)	1.8 (0.077)
Weight (kg) (*p* = 0.04)	76.5 (13.9)	79.5 (16.1)	73.6 (10.7)
BMI (*p* < 0.001)	24.7 (3.6)	26.1 (4.1)	23.4 (2.2)
Max power (watt) (*p* < 0.001)	260.1 (64.3)	231.4 (61.1)	288.7 (54.1)
VO_2_ (mL/min) (*p* < 0.001)	2822.8 (771)	2526.4 (755)	3119.2 (673)
VO_2_ per kg Body Weight (ml/min/kg) (*p* < 0.001)	37.2 (8.6)	32.0 (7.1)	42.4 (6.7)
1. Seismofit^®^ (mL/min/kg) (*p* < 0.001)	37.5 (8.1)	33.7 (7.8)	41.4 (6.5)
2. Seismofit^®^ (mL/min/kg) (*p* < 0.001)	37.3 (7.8)	33.6 (7.6)	40.9 (6.22)
Mean 1. & 2. Seismofit^®^ (mL/min/kg) (*p* < 0.001)	37.4 (7.9)	33.7 (7.7)	41.2 (6.3)
Post exhaustion Seismofit^®^ (mL/min/kg) (*p* < 0.001)	33.8 (7.09)	30.9 (7)	36.6 (6.03)
FRIENDS study (mL/min/kg) (*p* < 0.001)	32.5 (6.8)	30.1 (6.5)	34.9 (6.3)
ASCM MaxWatt (mL/min/kg) (*p* < 0.001)	44.2 (8.6)	38.8 (7.1)	49.7 (6.4)
Heart rate 1. Seismofit^®^ (BPM) (*p* < 0.001)	60.1 (9.3)	63.3 (9.6)	56.9 (7.8)
Heart rate 2. Seismofit^®^ (BPM) (*p* = 0.017)	60.5 (9.4)	62.8 (9.1)	58.2 (9.1)
Heart rate Post exhaustion Seismofit^®^ (BPM) (*p* = 0.44)	82.0 (10.5)	82.9 (10.2)	81.2 (10.8)

Continuous variables mean (SD) and categorical variables n (%). BPM: Beats per minute. Active lifestyle was defined as at least 5 h of exercise per week.

**Table 2:** Estimation performance of the Seismofit^®^, FRIENDS Study algorithm, and ACSM MaxWatt algorithm.

	1. Seismofit^®^	2. Seismofit^®^	Average 1. & 2	Post ex. Seismofit^®^	FRIENDS Study	ACSM MaxWatt
n	93	93	92	91	94	92
MAPE (%)	11.4 [9.74, 13%]	11.2 [9.59, 12.9%]	11.1 [9.5, 12.8%]	13.8 [12, 15.6%]	15.2 [13.1, 17.4%]	20.6 [18.4, 22.7%]
r	0.832 [0.76, 0.89]	0.826 [0.75, 0.88]	0.834 [0.76, 0.89]	0.769 [0.67, 0.84]	0.752 [0.65, 0.83]	0.947 [0.92, 0.96]
SEE (mL/min/kg)	4.9	4.9	4.9	6.6	7.4	7.7
Bias (mL/min/kg)	0.24 [−0.77, 1.2]	0.024 [−0.99, 1]	0.05 [−0.95, 1.1]	−3.6 [−4.7, −2.4] *	−4.7 [−5.8, −3.5] *	7.1 [6.5, 7.7] *
Upper LoA (mL/min/kg)	9.8	9.7	9.5	7.3	6.5	12.6
Lower LoA (mL/min/kg)	−9.3	−9.6	−9.4	−14.4	−15.8	1.6

Estimated value [95% confidence interval of estimate], n: number of subjects included in the analysis, MAPE: Mean Average Percentage Error, r: Pearson correlation coefficient, SEE: Standard Error of Estimate, LoA: Limits of Agreement. * Bias significantly different from zero.

**Table 3:** Estimation performance of the Seismofit^®^ in subgroups. Analysis was conducted using the average Seismofit^®^ score.

	n	Bias (mL/min/kg)	MAPE (%)	r	SEE (mL/min/kg)
All	92	0.05 [−0.95, 1.1]	11.1 [9.5, 12.8]	0.834 [0.76, 0.89]	4.9
Activity level					
Sedentary lifestyle	46	1.5 [0.25, 2.8] *	12.4 [9.66, 15.2]	0.831 [0.71, 0.9]	4.7
Active lifestyle	46	−1.4 [−2.9, −0.0026] *	9.8 [8.08, 11.6]	0.719 [0.54, 0.84]	5.1
Sex					
Female	48	−0.4 [−1.7, 0.88]	11.0 [8.99, 13.1]	0.801 [0.67, 0.88]	4.4
Male	44	0.5 [−1.1, 2.1]	11.3 [8.57, 13.9]	0.748 [0.58, 0.85]	5.4
Fitness level (VO_2MAX_)					
Low < 30 mL/kg/min	19	2.7 [0.78, 4.6] *	15.2 [9.69, 20.7]	0.694 [0.35, 0.87]	5.0
Moderate 30–45 mL/kg/min	55	0.3 [−0.93, 1.5]	10.0 [8.19, 11.7]	0.673 [0.5, 0.8]	4.5
Good > 45 mL/kg/min	18	−3.4 [−5.9, −0.89] *	10.4 [7.48, 13.3]	0.359 [−0.13, 0.71]	6.4
BMI					
Normal weight: <25 kg/m^2^	59	−0.13 [−1.4, 1.1]	9.9 [8.34, 11.4]	0.788 [0.67, 0.87]	4.8
Overweight: 20–29.9 kg/m^2^	27	0.7 [−1.4, 2.8]	13.6 [9.39, 17.9]	0.773 [0.56, 0.89]	5.4
Obese: ≥30 kg/m^2^	6	−1.0 [−5.6, 3.5]	12.4 [2.87, 21.9]	0.865 [0.18, 0.99]	5.0
Age					
<50 years	53	0.3 [−1.1, 1.7]	10.5 [8.4, 12.6]	0.794 [0.67, 0.88]	5.1
>50 Years	39	−0.3 [−1.7, 1.2]	12 [9.32, 14.7]	0.842 [0.72, 0.91]	4.6

Estimated value [95% confidence interval of estimate], n: number of subjects included in the analysis, MAPE: Mean Average Percentage Error, r: Pearson correlation coefficient, SEE: Standard Error of Estimate. Active lifestyle was defined as at least 5 h of exercise per week. * Significant bias (*p* < 0.05).

## Data Availability

The data presented in this study are available on request from the corresponding author due to privacy and data protection.

## Appendix A

**Table A1:** Correlation between the variables included in the Seismofit^®^ estimation algorithm and VO_2MAX_.

	All	Male	Female
**Pre-exercise 1. measurement**			
Sex (Male = 1)	0.49		
Age (Years)	−0.314	−0.409	−0.374
Weight (kg)	−0.177	−0.607	−0.549
Height (cm)	0.357	−0.0704	0.12
RR (s)	0.421	0.273	0.412
Dia_Peak to Peak_	0.423	0.585	0.58
Sys_Spectrum_	0.371	0.414	0.456
Dia_Morphology_	0.603	0.486	0.317
**Pre-exercise 2. measurement**			
Sex (Male = 1)	0.47		
Age (Years)	−0.321	−0.43	−0.377
Weight (kg)	−0.188	−0.595	−0.555
Height (cm)	0.341	−0.0869	0.12
RR (s)	0.304	0.181	0.344
Dia_Peak to Peak_	0.442	0.59	0.61
Sys_Spectrum_	0.397	0.5	0.446
Dia_Morphology_	0.577	0.442	0.29
**Post-exercise measurement**			
Sex (Male = 1)	0.43		
Age (Years)	−0.299	−0.429	−0.356
Weight (kg)	−0.193	−0.597	−0.569
Height (cm)	0.338	−0.0956	0.25
RR (s)	0.0331	0.0472	0.0558
Dia_Peak to Peak_	0.165	0.412	0.457
Sys_Spectrum_	0.31	0.389	0.371
Dia_Morphology_	0.493	0.179	0.394
